# Effect of peer support interventions on cardiovascular disease risk factors in adults with diabetes: a systematic review and meta-analysis

**DOI:** 10.1186/s12889-018-5326-8

**Published:** 2018-03-23

**Authors:** Sonal J. Patil, Todd Ruppar, Richelle J. Koopman, Erik J. Lindbloom, Susan G. Elliott, David R. Mehr, Vicki S. Conn

**Affiliations:** 1 Curtis W. and Ann H. Long Department of Family and Community Medicine, University of Missouri, MA306 Medical Sciences Building, DC032.00, Columbia, MO 65212 USA; 2 College of Nursing, Rush University Medical Center, Chicago, IL USA; 3 Sinclair School of Nursing, University of Missouri, Columbia, MO USA

**Keywords:** Peer support, Diabetes, Cardiovascular health metrics, Self-management, Blood pressure, Physical activity, Behavioral counseling

## Abstract

**Background:**

Peer support by persons affected with diabetes improves peer supporter’s diabetes self-management skills. Peer support interventions by individuals who have diabetes or are affected by diabetes have been shown to improve glycemic control; however, its effects on other cardiovascular disease risk factors in adults with diabetes are unknown. We aimed to estimate the effect of peer support interventions on cardiovascular disease risk factors other than glycemic control in adults with diabetes.

**Methods:**

We conducted a systematic review and meta-analysis of randomized controlled trials comparing peer support interventions to a control condition in adults affected by diabetes that measured any cardiovascular disease risk factors [Body Mass Index, smoking, diet, physical activity, cholesterol level, glucose control and blood pressure]. Quality was assessed by Cochrane’s risk of bias tool. We calculated standardized mean difference effect sizes using random effects models.

**Results:**

We retrieved 438 citations from multiple databases including OVID MEDLINE, Cochrane database and Scopus, and author searches. Of 233 abstracts reviewed, 16 articles met inclusion criteria. A random effects model in a total of 3243 participants showed a positive effect of peer support interventions on systolic BP with a pooled effect size of 2.07 mmHg (CI 0.35 mmHg to 3.79 mmHg, *p* = 0.02); baseline pooled systolic blood pressure was 137 mmHg. There was a non-significant effect of peer support interventions on diastolic blood pressure, cholesterol, body mass index, diet and physical activity. Cardiovascular disease risk factors other than glycemic control outcomes were secondary outcomes in most studies and baseline values were normal or mildly elevated. Only one study reported smoking outcomes.

**Conclusions:**

We found a small (2 mmHg) positive effect of peer support interventions on systolic blood pressure in adults with diabetes whose baseline blood pressure was on average minimally elevated. Additional studies need to be conducted to further understand the effect of peer support interventions on high-risk cardiovascular disease risk factors in adults with diabetes.

**Electronic supplementary material:**

The online version of this article (10.1186/s12889-018-5326-8) contains supplementary material, which is available to authorized users.

## Background

In the last decade, several trials have looked at the effects of peer support interventions in adults with diabetes; several of these have looked at changes in cardiovascular disease risk factors. Diabetes increases cardiovascular disease (CVD) risk by twofold, and, in patients with type 2 diabetes, cholesterol and blood pressure lowering appear more important than glucose lowering in reducing cardiovascular events [1, 2]. The American Heart Association (AHA) introduced the concept of cardiovascular health characterized by seven ideal metrics namely Body Mass Index (BMI), smoking, diet, physical activity, cholesterol level, blood pressure and glucose levels, to improve the cardiovascular health of all Americans while reducing deaths from CVD and strokes [3, 4]. These metrics are also known as Life’s Simple 7 [3]. Several studies and a 2016 systematic review of population studies found an inverse relationship between an increasing number of ideal AHA cardiovascular health metrics and all-cause mortality, CVD-related mortality, incidence of stroke, and incidence of non-CVD outcomes such as cancer, depression and cognitive decline [5–7]. A 2014 systematic review for the U.S. Preventive Services Task Force (USPSTF) showed that intensive behavioral counseling for diet and physical activity in people with CVD risk factors resulted in improvements in cholesterol, blood pressure and fasting glucose levels [8]. Peer support interventions could be a sustainable community-based intervention for behavioral counseling in people with CVD risk factors.

A 2010 systematic review of peer-support intervention trials for individuals with heart disease did not find studies reporting clinical outcomes or CVD risk factor outcomes [9]. However, there have been several randomized controlled trials (RCTs) of peer support interventions in people affected by diabetes looking at diabetes and cardiovascular disease risk factor outcomes. Several systematic reviews have shown improved glycemic outcomes with peer support interventions [10–12]; however, to date, there have been no systematic reviews and meta-analyses synthesizing the effect of peer support interventions on cardiovascular disease risk factors other than glycemic control in adults with diabetes. Consequently, we conducted a systematic review and meta-analysis of randomized controlled trials to assess the effectiveness of peer support interventions on cardiovascular disease risk factors other than glucose control in adult patients with diabetes compared to otherwise similar care.

We have defined peer support as support from a person who has knowledge from their own experiences with a chronic condition, which in our study is diabetes. This definition is from of the American Academy of Family Physicians (AAFP) Peers for Progress program [13]. Being a peer supporter empowers patients to improve their diabetes self-management skills while incorporating personal experiences to empathize with other affected individuals [14]. A qualitative study of peer leaders in a study that compared peer-delivered versus professionally delivered hypertension self-management education showed that peer leaders noted improvements in their own systolic blood pressure, hypertension knowledge, pedometer use and fruit and vegetable intake [15]. We chose the AAFP Peers for Progress program definition of peer support because engaging individuals affected with chronic conditions in intervention delivery to people affected by similar conditions may further enhance patient engagement in self-management behaviors.

## Methods

We have used the PRISMA statement and reporting system for reporting the findings of our systematic review [16].

### Search strategy and study selection

Randomized controlled trials of peer-support interventions compared to otherwise similar care in adult patients with diabetes that measured any of the cardiovascular disease risk factors (BMI, smoking, diet, physical activity, cholesterol level, blood pressure and glucose levels) as primary or secondary outcomes were included. Studies with adult participants affected with any type of diabetes were included. We used the AAFP Peers for Progress definition of peer support which is support from a person with diabetes or a person affected by diabetes [13]. Studies were excluded if the intervention group received any additional care other than peer-delivered intervention compared to control group. Studies with peer support intervention facilitators where the facilitators were professionals or not affected by diabetes were excluded. Studies comparing a peer-delivered intervention to an identical intervention delivered by other health professionals were excluded as well. The only difference between the intervention and the control group had to be a peer-delivered intervention. We made this choice because our goal was to understand the added value of peer support to usual care rather than how peer support compared to other support interventions.

### Data sources

To ensure a comprehensive review, we did separate searches for papers involving peer support and diabetes and peer support and cardiovascular risk factors. We initially searched multiple databases, including Ovid MEDLINE, the Cochrane Central Register of Controlled Trials, Scopus, CINAHL, PsycINFO, OCLC First Search, and a few others for English and non-English articles from January 1960 through November 2015. Groups of search terms included diabetes mellitus; RCT; peer support, promotora, peer educator, peer coach; HbA1C. We additionally, searched Ovid Medline and Scopus for English articles from January 1990 to August 2016 with additional search terms. Groups of search terms for the second search included peer health, peer support, peer counselor, peer adviser, peer coach, promotora; hypertension, high blood pressure, cardiovascular, cardiac, heart, vascular, arteries, veins; RCT. To find additional studies, we also conducted author searches for authors with known expertise in peer-support research, and we searched references in published articles and the WHO statement on peer support in diabetes management [17]. We also reviewed relevant recent systematic reviews of interventions targeting lifestyle changes conducted in adults affected with diabetes to check for any additional missed studies [18–21]. Two reviewers (S.P. and R.J.K) independently screened all the citations, and two additional reviewers (T.R. and V.C.) confirmed their eligibility.

### Quality assessment

Study quality was assessed using the Cochrane collaboration’s risk of bias tool and checked for inter-rater comparability by two authors (S.P. and E.L.) [22].

### Data extraction

One author (S.P.) created a codebook to include all variables of interest, which was reviewed by three other authors (E.L., T.R., and V.C.) before data extraction. Data were extracted independently by two authors (S.P. and E.L.). One additional author (T.R.) confirmed the extraction accuracy of numerical outcome data. We extracted data on study setting, intervention characteristics and multiple participant and peer characteristics. Blood pressure, cholesterol levels, BMI, physical activity, diet and smoking means and measures of statistical variation were extracted at baseline and study conclusion.

### Data synthesis and analysis

Statistical analysis was performed using Comprehensive Meta-analysis Software version 3 (Biostat Inc., Englewood, NJ). The standardized mean difference in outcomes between peer support intervention group and control group was calculated using the DerSimonian and Laird random-effects model [23]. The standardized mean difference effect size expresses the difference in means between intervention and control groups in terms of their shared standard deviation [24]. We used change from baseline values for analysis and assumed a correlation coefficient of 0.5 between initial and final values [25]. We considered a *p* value of < 0.05 as statistically significant and calculated 95% confidence intervals (CI). Heterogeneity was evaluated by the Q statistic with a *p*-value of < 0.10 indicating significant heterogeneity. The proportion of unexplained heterogeneity was described using the I^2^ statistics with I^2^ values > 75% indicating significant heterogeneity, I^2^ > 50% indicating substantial heterogeneity, and I^2^ > 30% indicating moderate heterogeneity [26]. We planned moderator analyses when ten or more studies were measuring the specified outcome. We conducted moderator analyses using meta-regression. Funnel plots and Eggers regression were used to assess publication bias [27].

## Results

### Study selection

**Fig. 1:**
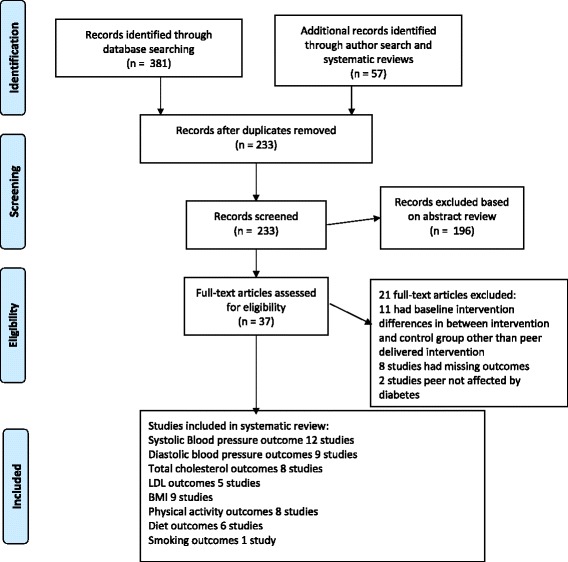
Results of literature search

We retrieved 438 citations from database searches and database author searches. We examined 37 full articles after removing duplicates and reviewing 233 abstracts. Despite our broader search, all retrieved citations were in English. Of the 37 full articles, 16 studies met the inclusion criteria. All disagreements were discussed and resolved to achieve 100% consensus for article selection (with four raters) and data abstraction (with three raters). Figure 1 shows the flow diagram of the literature search [28]. Of the 16 studies, 12 studies reported systolic BP outcomes, nine studies reported diastolic BP, eight studies reported total cholesterol, five reported LDL cholesterol outcomes, nine studies reported BMI outcomes, eight studies reported physical activity outcomes, six studies reported diet outcomes and one study reported smoking outcomes. All but one study ranged from 6 weeks to 12 months in duration of intervention and follow-up. Table 1 shows study characteristics.

**Table 1:** Descriptive summary of included trial characteristics

Study name, year published	Study duration in months	Sam-ple size	Country, Population	Baseline care for intervention and control group	Intervention	Cardiovascular health metrics measured
Keyserling, 2002 [34]	12	133	U.S: African-American women	Four individual counseling visits with nutritionist	Peer-delivered three group sessions and monthly telephone calls from a peer-counselor for 12 months. Peer supporter training duration: 16 h	Total cholesterol Weight Diet: calories from saturated fat, dietary cholesterol, total energy intake Physical activity measured by accelerometer
Lorig, 2008 [44]	6	417	U.S: Spanish-speaking patients	Usual care	2.5 h weekly sessions led by two peer leaders for 6 weeks. Peer supporter training duration: 24 h	Physical activity: exercise minutes per week
Lorig, 2009 [45]	6	345	US: White, non-Hispanic 67%	Usual care	Peer leader delivered 2.5 h weekly sessions for 6 weeks. Peer supporter training duration: 24 h	Physical activity: exercise minutes per week Weight
Cade, 2009 [29]	12	207	U.K: White, European origin 95%	Usual care	2-h education sessions per week for 7 weeks by peer educators. Residential peer supporter training mentioned without hours	Systolic Blood pressure Diastolic Blood pressure Total Cholesterol BMI Diet: energy, protein, fat, fiber and fruit and vegetable intake
Heisler, 2010 [46]	6	244	U.S.: Male veterans white non-Hispanic 82%	Usual care	Weekly calls encouraged between peer partners. Optional 1.5 h face-to-face sessions at 1, 3, and 6 months. Peer supporter training duration: 1.5 h	Systolic Blood pressure Diastolic Blood pressure
Philis-Tsimikas, 2011 [47]	10	207	US.: Mexican Americans	Usual care	2.5-h weekly education sessions by peer educators for 8 weeks. Peer supporter training duration: 40 h	Systolic Blood pressure Diastolic Blood pressure Total Cholesterol LDL cholesterol BMI
Smith, 2011 [30]	24	388	Republic of Ireland: 50% of the population was low income Ireland general demographics 94.3% White non-Hispanic	Usual care	Peer-supporter facilitated sessions over 2 years; at month one, month two, and every 3 months thereafter (total nine sessions). Peer supporter training duration: 6 h	Systolic Blood pressure Total Cholesterol BMI Smoking Summary of self-care activities (SDSCA): scale to measure diet and physical activity
Gagliardino, 2013 [48]	12	198	Argentina: Hispanic, Non-minority in country of residence	Five 90-120 min sessions with diabetes educators	Initially weekly peer educator sessions of 90–120 min for 4 weeks; one at 6 months followed by weekly calls for 6 months then biweekly calls for 3 months. Additional face-to-face visits with peer supporters were scheduled every second month if specific issues warranted. Peer supporter training duration: 3-day training course	Systolic Blood pressure Diastolic Blood pressure Total Cholesterol BMI
Siminerio, 2013 [31]	6	68	U.S.: > 80% White non-Hispanic ethnicity	Certified diabetes educator delivered diabetes self- management education for 6 weeks	Monthly peer calls for 6 months Peer supporter training duration: 2–3 h	Systolic Blood pressure Diastolic Blood pressure Total Cholesterol LDL cholesterol BMI Summary of self-care activities (SDSCA): scale to measure diet and physical activity
Thom, 2013 [49]	6	299	U.S: Hispanic 46.65% and African American 31.25%	Usual care	Telephone contacts with peers at least twice a month and 2 or more in-person contacts in 6 months. Peer supporter training duration: 36 h	Systolic Blood pressure LDL cholesterol BMI
Chan, 2014 [32]	12	628	China: 100% Chinese speaking	Two-hour nurse-led empowerment class and personalized, comprehensive assessment report to all participants at baseline and follow-up	Peer supporter phone calls biweekly for 3 months, then monthly for 3 months, and then 1 call every other month for 6 months; anticipated 15 min per call Peer supporter training duration: 32 h	Systolic Blood pressure Diastolic Blood pressure Total Cholesterol LDL cholesterol BMI Weight; Waist Summary of self-care activities (SDSCA): scale to measure diet and physical activity
Simmons, 2015 [50]	12	644	England: Cluster randomized factorial design > 90% white,non-Hispanic	Usual care	Peer-led group education sessions once a month for at least 5 months and telephone/email for 1:1 counseling Peer supporter training duration: 17.5 h	Systolic Blood pressure Diastolic Blood pressure Total Cholesterol Weight; Waist
Safford, 2015 [51]	15	424	US: Cluster randomized trial > 90% African Americans	One diabetes group education class with 5 min of individual counseling with diabetes report card.	Initial 45–60 min in-person or telephone get to know session with peer supporter followed by weekly calls for 2 months followed by monthly calls for 8 months Peer supporter training duration: 12 h	Systolic Blood pressure LDL cholesterol BMI
Ayala, 2015 [33]	12	336	US: Predominantly Hispanic	Usual care	8 telephone or in-person contacts with peer supporter in first 6 months, then as needed contacts in the last 6 months. 92% of participants had telephone contacts. Peer supporter training duration: 40–50 h	Systolic Blood pressure Diastolic Blood pressure SDSCA to measure diet and physical activity
McGowan, 2015 [52]	12	361	Canada: Ethnicities not mentioned	Usual care	Peer-led self- management programs with varying components: weekly meetings for 6 weeks Peer supporter training duration: 24 h	Systolic Blood pressure Diastolic Blood pressure LDL cholesterol BMI Weight
Sazlina, 2015 [53]	8	46	Malaysia: Asian, urban primary care clinic in Selangor, Malaysia	Three sessions providing personalized feedback about participant’s physical activity patterns	Peer supporters had three face-to-face and three telephone contacts over the 12 weeks. Peers motivated and provided support to the participants to walk regularly Peer supporter training duration: 2-day training program	Systolic Blood pressure Diastolic Blood pressure LDL cholesterol BMI Physical activity measured by pedometer

### Risk of Bias and publication Bias

Most included studies mentioned methods of randomization and allocation concealment, but none of the studies blinded participants. The risk of bias assessment for included studies is shown in Additional file 1. The outcomes and the quality of the studies were not interrelated. For all the outcomes examined with a meta-analysis, Funnel plots and Egger regression tests did not show any publication bias.

### Peer support effect on blood pressure

**Fig. 2:**
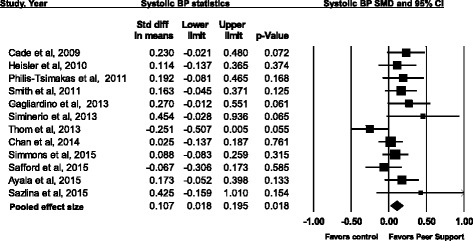
Effect of peer support interventions on systolic blood pressure in adults with diabetes. SMD = Standardized mean difference; Systolic BP = systolic blood pressure. *I*^*2*^ 35.75%, p for heterogeneity = 0.113

For a total of 3243 participants, the overall pooled effect of peer-support interventions on systolic BP was a standardized mean difference of 0.107 (CI 0.018 to 0.195; *p* = 0.02, *I*^*2*^ 34.47%), which translated to improvement in systolic BP of 2.07 mmHg (CI 0.35 to 3.79) where the pooled mean baseline systolic BP was 137 mmHg (Fig. 2 and Table 2). Meta-regression did not show any interaction between the baseline systolic BP and effect sizes (See Additional file 2). However, in all but one study, baseline systolic BP was in the range of 121 to 144. In one study, the average baseline systolic BP was 152 [29]. A funnel plot and the Egger regression test did not show any publication bias (See Additional file 3). Meta-regression did not show any interaction between study duration and effect sizes (See Additional file 4).

**Table 2:** Results of peer support intervention effects on cardiovascular health outcomes

Outcome	Number of studies	Pooled effect size (SMD)^a^	95% CI	Baseline pooled mean	Pooled effect size in original metric	95% CI in original metric	p-value for SMD	*I*^*2*^ (%)	Publication bias: p-value of Egger Regression test
Systolic Blood pressure	12	0.107	0.018 to 0.195	137 mm hg	2.07 mm hg	0.35 to 3.79 mm hg	0.02	34.47	0.15
Diastolic Blood Pressure	9	0.039	−0.086 to 0.164	77 mm hg	0.42 mm hg	−0.94 to 1.79 mm hg	0.5	49.08	0.6
Total Cholesterol	8	0.058	−0.022 to 0.138	181 mg/dL	2.61 mg/dL	−0.99 to 6.21 md/dl	0.1	0.00	0.2
LDL cholesterol	5	−0.008^b^	−0.124 to 0.109	107.85 mg/dl	- 0.28 mg/dl	−4.41 to 3.87 mg/dl	0.8	8.47	0.2
BMI	9	0.017	−0.071 to 0.104	31.27	0.11	−0.48 to 0.7	0.7	0.00	0.9
Physical activity	8	0.019	−0.068 to 0.106	N/A^c^	N/A^c^		0.6	0.00	0.07

^a^pooled effect in terms of standardized mean difference (SMD) which is the difference in means between intervention and control participants in terms of their standard deviations

^b^negative sign indicates intervention effect favored control group

^c^Not Applicable since physical activity could not be converted to original metric due to diverse measurement scales used in included studies

Diet and Smoking outcomes could not be summarized quantitatively. No significant differences were observed for diet and smoking outcomes between groups

There was no significant effect of peer support interventions on diastolic blood pressure (Table 2 and Additional file 5).

### Peer support effect on cholesterol, BMI, physical activity, diet, and smoking

Meta-analysis of included studies did not show a significant pooled effect of peer support on total cholesterol, LDL-cholesterol, BMI, or physical activity. (Table 2 and See Additional file 6, Additional file 7, Additional file 8, Additional file 9). Physical activity was measured using varying instruments in included studies. Two studies measured physical activity with an accelerometer or pedometer, two studies used a physical activity scale measuring minutes of aerobic exercise per week, and four studies used the Summary of Diabetes Self-Care Activities (SDSCA) scale to measure physical activity (See Table 1). The effect of peer support on diet could not be summarized quantitatively due to varying dietary outcome measurements (e.g., total calories, calories from fat, or, fruit and vegetable intake). Four studies used the SDSCA to assess healthy diet and food and vegetable consumption in the past week [30–33]. One study utilized a series of three-day telephone-administered recalls of food intake, and another study used three-day food diaries and questionnaires [29, 34]. None of the studies showed significant differences in dietary intake between intervention and control groups. Only one study looked at the effect of a peer support intervention on smoking in adults with diabetes and did not show any significant difference between the peer support intervention group and the control group [30].

## Discussion

The CDC cost-effectiveness group has reported that intensified hypertension control in adults with diabetes leads to reduced costs and better health outcomes whereas intensified glucose and cholesterol control leads to increased costs and improved outcomes [35]. In this review, peer support interventions were associated with a small but statistically significant improvement in systolic blood pressure with low-moderate heterogeneity of results across studies despite the pooled baseline systolic BP being less than 140 mmHg. Despite baseline systolic blood pressures being < 140 in most subjects in our meta-analysis, the improvement in systolic BP is particularly relevant with new evidence and guidelines that set a lower BP target of < 130/80 in patients with diabetes [36]. The effect size of 2 mmHg improvement is consistent with improvements seen with behavioral counseling interventions to promote a healthy lifestyle and reduce cardiovascular risk factors in a systematic review for the USPSTF [37]. Even though this improvement in systolic BP is small, at the population level it would be clinically relevant if sustained over time. For example, a 2 mm decrease in systolic blood pressure is associated with mortality reductions of 6% due to stroke, 4% due to coronary heart disease and 3% in total mortality [38]. Peer-delivered interventions might be a feasible alternative for hypertension and diabetes self-management education in places with limited professional resources.

We did not find any significant effects of peer support interventions on any other cardiovascular disease risk factors except for systolic BP. One potential reason might be that peer intervention trials in patients affected with diabetes frequently targeted glucose control while participants had normal or only mildly abnormal baseline values of blood pressure, cholesterol, and BMI. Peer support interventions that specifically target CVD risk factors besides glycemic control in adults with diabetes need to be designed and tested. Even though the results were statistically non-significant, there was a positive effect of peer support interventions on total cholesterol with low heterogeneity of results noted across studies. Our meta-analysis may not have been powered sufficiently to detect a difference in total cholesterol between the peer intervention group and the control group, especially since the baseline total cholesterol was completely normal. Following completion of a study, consideration of confidence intervals is the most appropriate way to view the potential for a clinically important difference with a larger study [39]. For BMI we found an effect size estimate of 0.11 with a confidence interval that includes the null effect, however, with an upper confidence limit for BMI of 0.7, a larger study might demonstrate an important difference. Similarly for diastolic BP we found an effect size of 0.42 mmHg, but with an upper confidence limit of 1.79, a larger study might also demonstrate an important difference. For total cholesterol, we found a difference of 2.61 mg/dl in total cholesterol with an upper confidence limit of 6.21 mg/dl. For perspective, USPSTF recommendations on behavioral counseling to prevent cardiovascular disease in adults were based on improvement in BMI of at least 0.3, improvement in diastolic BP of at least 1.1 mmHg and improvement in total cholesterol of at least 6.56 mg/dl [40]. Further larger studies are needed potentially to detect improvements in BMI and diastolic BP of at least these levels; however, clinically significant improvements in total cholesterol might be challenging to achieve.

In a few studies not limited to adults with diabetes, peer support intervention effects on cardiovascular disease risk factors have shown promise. Recently, a Spanish randomized controlled trial studying the effect of peer support interventions on self-control of unhealthy behaviors in individuals at risk of cardiovascular disease showed improvement in cardiovascular health after 1 year, specifically in smoking cessation [41]. Another study of peer-led education for secondary prevention among predominantly minority stroke survivors led to improved systolic BP control, but no difference in control of cholesterol [42]. Peer support interventions may be a feasible way to initiate and maintain cardiovascular health in large populations; however further studies in persons with higher baseline cardiovascular risk factors are required.

### Limitations

Because we evaluated peer support intervention trials in adults affected by diabetes, most of the included trials may have targeted improving diabetes control rather than overall cardiovascular health. Additionally, we found few studies reporting some outcomes, specifically LDL, and smoking outcomes; this resulted in lower power to detect significant differences between groups. Moreover, most of the studies included in our review had an intervention duration between 6 weeks to 12 months (only one study included a longer duration); it would be challenging to demonstrate a positive effect of behavioral interventions over such a short duration, especially when most study participants had normal or mildly abnormal baseline values of cardiovascular disease risk factors. Another limitation of our study was that we used the outcomes at study conclusion, therefore, we have lumped changes between baseline and multiple different follow-up times ranging from 6 months to 24 months. However, in a meta-regression with our systolic blood pressure results, we did not find an interaction of effect size and intervention duration. Most studies measured diet and physical activity using the SDSCA scale which is a self- report instrument that has been validated using multiple methods of self-report [43]. Similarly, only two studies measured physical activity using accelerometer and pedometer data while six studies used self-report scales to measure physical activity. We acknowledge there is always the risk of recall bias and social desirability with self-report measures. Blinding is not possible in peer support intervention trials since a peer needs to deliver the intervention to the participants; however, only two of all the included studies in our review blinded outcomes assessors. One additional study limitation is the possibility we missed studies reporting cardiovascular disease risk factors in adults with diabetes.

## Conclusions

In adults affected with diabetes, peer support interventions lead to a small improvement in systolic blood pressure when mean baseline systolic blood pressure levels were just minimally elevated according to new AHA guideline standards. The effectiveness of peer support interventions for improving overall cardiovascular disease risk factors needs to be further studied in adults affected with diabetes who have high-risk baseline cardiovascular disease risk factors.

## Additional files

Additional file 1: Quality assessment of the included studies with Cochrane’s risk of bias tool. (DOCX 25 kb)

Additional file 2: Regression of standardized difference in means of included studies on baseline systolic blood pressure. (PDF 5 kb)

Additional file 3: Funnel plot of standard error by standardized difference in means for systolic blood pressure. (PDF 5 kb)

Additional file 4: Regression of standardized difference in means of included studies on Study duration. (PDF 84 kb)

Additional file 5: Effect of peer support interventions on diastolic blood pressure in adults with diabetes. SMD = Standardized mean difference; Diastolic BP = systolic blood pressure. *I*^*2*^ 49.07%, p for heterogeneity = 0.05. (PDF 8 kb)

Additional file 6: Effect of peer support interventions on cholesterol in adults with diabetes. SMD = standardized mean difference; *I*^*2*^ 0.00%, p for heterogeneity = 0.7. (PDF 8 kb)

Additional file 7: Effect of peer support interventions on LDL cholesterol in adults with diabetes. SMD = standardized mean difference; *I*^*2*^ 0.00%, p for heterogeneity = 0.819. (PDF 8 kb)

Additional file 8: Effect of peer support interventions on BMI in adults with diabetes. SMD = standardized mean difference; BMI = Body Mass Index; *I*^*2*^ 0.00%, p for heterogeneity = 0.9. (PDF 86 kb)

Additional file 9: Effect of peer support interventions on physical activity in adults with diabetes. SMD = standardized mean difference; *I*^*2*^ 0.00%, p for heterogeneity = 0.47. (PDF 8 kb)

## Abbreviations

AAFP: American academy of family physicians
AHA: American Heart Association
BMI: Body mass index
BP: Blood pressure
CDC: Centers for disease control and prevention
CI: Confidence intervals
CVD: Cardiovascular disease
HbA1C: Hemoglobin A1C
LDL cholesterol: Low-density lipoprotein cholesterol
RCTs: Randomized controlled trials
SDSCA: Summary of diabetes self-care activities scale
SMD: Standardized mean difference
USPSTF: U.S. preventive services task force
WHO: World Health Organization
