# Supplementary material for: Effect of peer support interventions on cardiovascular disease risk factors in adults with diabetes: a systematic review and meta-analysis
Source: BMC Public Health. 2018 Mar 23;18:398. doi: 10.1186/s12889-018-5326-8 (PMC5865386; doi:10.1186/s12889-018-5326-8)
Supplement: Supplementary file 1 — Quality assessment of the included studies with Cochrane’s risk of bias tool. (DOCX 25 kb) [file 12889_2018_5326_MOESM1_ESM.docx]

| Author, year published  Additional File 1: Quality assessment of the included studies with Cochrane’s risk of bias tool | **Adequate Sequence Generation** | **Allocation Concealment** | **Blinding of Investigators and/or Outcome Assessors** | **Complete Outcome Data** |
| --- | --- | --- | --- | --- |
| Keyserling et al, 2002 [1] | YES | YES | NO | YES |
| Lorig et al, 2008 [2] | YES | YES ^a^ | NO | YES |
| Lorig et al 2009 [3] | YES | YES ^a^ | NO | YES |
| Cade et al, [4] | YES? | ?NO | ?NO | YES |
| Heisler et al, 2010 [5] | YES | YES | YES | YES |
| Philis-Tsamakas et al, 2011 [6] | YES | YES ^a^ | NO ^a^ | YES |
| Smith et al, 2011[7] | YES | YES | NO | YES |
| Gagliardino et al, 2013[8] | YES? | ?NO | ?NO | ?NO |
| Siminerio et al, 2013[9] | YES | NO ^a^ | NO | YES |
| Thom et al, 2013[10] | YES | YES | NO ^a^ | YES |
| Chan et al, 2014 [11] | YES | YES | ?NO | YES |
| Simmons et al, 2015 [12] | YES | YES | YES | YES |
| Safford et al, 2015 [13] | YES | YES | NO | YES |
| Ayala et al, 2015 [14] | YES | ?NO | ?NO | YES |
| McGowan et al, 2015 [15] | YES | YES | NO | YES |
| Sazlina et al, 2015 [16] | YES | YES | NO | YES |

Footnotes:

Yes indicates low risk of bias; Yes? Likely low risk of bias but no details on sequence generation available from article and author; No indicates high risk of bias; ?No is not reported or no response from author.

a: author response to information requests

Selective outcome reporting risk was not possible to assess. There were no significant other biases noted in the included studies.

None of the trials blinded participants.

Reference List

1. Keyserling TC, Samuel-Hodge CD, Ammerman AS, et al (2002) A randomized trial of an intervention to improve self-care behaviors of African-American women with type 2 diabetes: impact on physical activity. Diabetes Care 25: 1576-1583

2. Lorig K, Ritter PL, Villa F, Piette JD (2008) Spanish diabetes self-management with and without automated telephone reinforcement: two randomized trials. Diabetes Care 31: 408-414

3. Lorig K, Ritter PL, Villa FJ, Armas J (2009) Community-based peer-led diabetes self-management: a randomized trial. Diabetes Educ 35: 641-651

4. Cade JE, Kirk SF, Nelson P, et al (2009) Can peer educators influence healthy eating in people with diabetes? Results of a randomized controlled trial. Diabet Med 26: 1048-1054

5. Heisler M, Vijan S, Makki F, Piette JD (2010) Diabetes control with reciprocal peer support versus nurse care management: a randomized trial. Ann Intern Med 153: 507-515

6. Philis-Tsimikas A, Fortmann A, Lleva-Ocana L, Walker C, Gallo LC (2011) Peer-led diabetes education programs in high-risk Mexican Americans improve glycemic control compared with standard approaches: a Project Dulce promotora randomized trial. Diabetes Care 34: 1926-1931

7. Smith SM, Paul G, Kelly A, Whitford DL, O'Shea E, O'Dowd T (2011) Peer support for patients with type 2 diabetes: cluster randomised controlled trial. BMJ 342: d715

8. Gagliardino JJ, Arrechea V, Assad D, et al (2013) Type 2 diabetes patients educated by other patients perform at least as well as patients trained by professionals. Diabetes Metab Res Rev 29: 152-160

9. Siminerio L, Ruppert KM, Gabbay RA (2013) Who can provide diabetes self-management support in primary care? Findings from a randomized controlled trial. Diabetes Educ 39: 705-713

10. Thom DH, Ghorob A, Hessler D, De VD, Chen E, Bodenheimer TA (2013) Impact of peer health coaching on glycemic control in low-income patients with diabetes: a randomized controlled trial. Ann Fam Med 11: 137-144

11. Chan JC, Sui Y, Oldenburg B, et al (2014) Effects of telephone-based peer support in patients with type 2 diabetes mellitus receiving integrated care: a randomized clinical trial. JAMA Intern Med 174: 972-981

12. Simmons D, Prevost AT, Bunn C, et al (2015) Impact of community based peer support in type 2 diabetes: a cluster randomised controlled trial of individual and/or group approaches. PLoS ONE 10: e0120277

13. Safford MM, Andreae S, Cherrington AL, et al (2015) Peer coaches to improve diabetes outcomes in rural Alabama: a cluster randomized trial. Ann Fam Med 13: S18-S26

14. Ayala GX, Ibarra L, Cherrington AL, et al (2015) Puentes hacia una mejor vida (Bridges to a better life): outcome of a diabetes control peer support intervention. Ann Fam Med 13: S9-S17

15. McGowan P (2015) The relative effectiveness of self-management programs for type 2 diabetes. Can J Diabetes 39: 411-419

16. Sazlina SG, Browning CJ, Yasin S (2015) Effectiveness of personalized feedback alone or combined with peer support to improve physical activity in sedentary older Malays with Type 2 Diabetes: a randomized controlled trial. Front Public Health 3: 178
