# Supplementary figures and images for: Effect of peer support interventions on cardiovascular disease risk factors in adults with diabetes: a systematic review and meta-analysis
Source: BMC Public Health. 2018 Mar 23;18:398. doi: 10.1186/s12889-018-5326-8 (PMC5865386; doi:10.1186/s12889-018-5326-8)

### Regression of Std diff in means on Baseline SBP

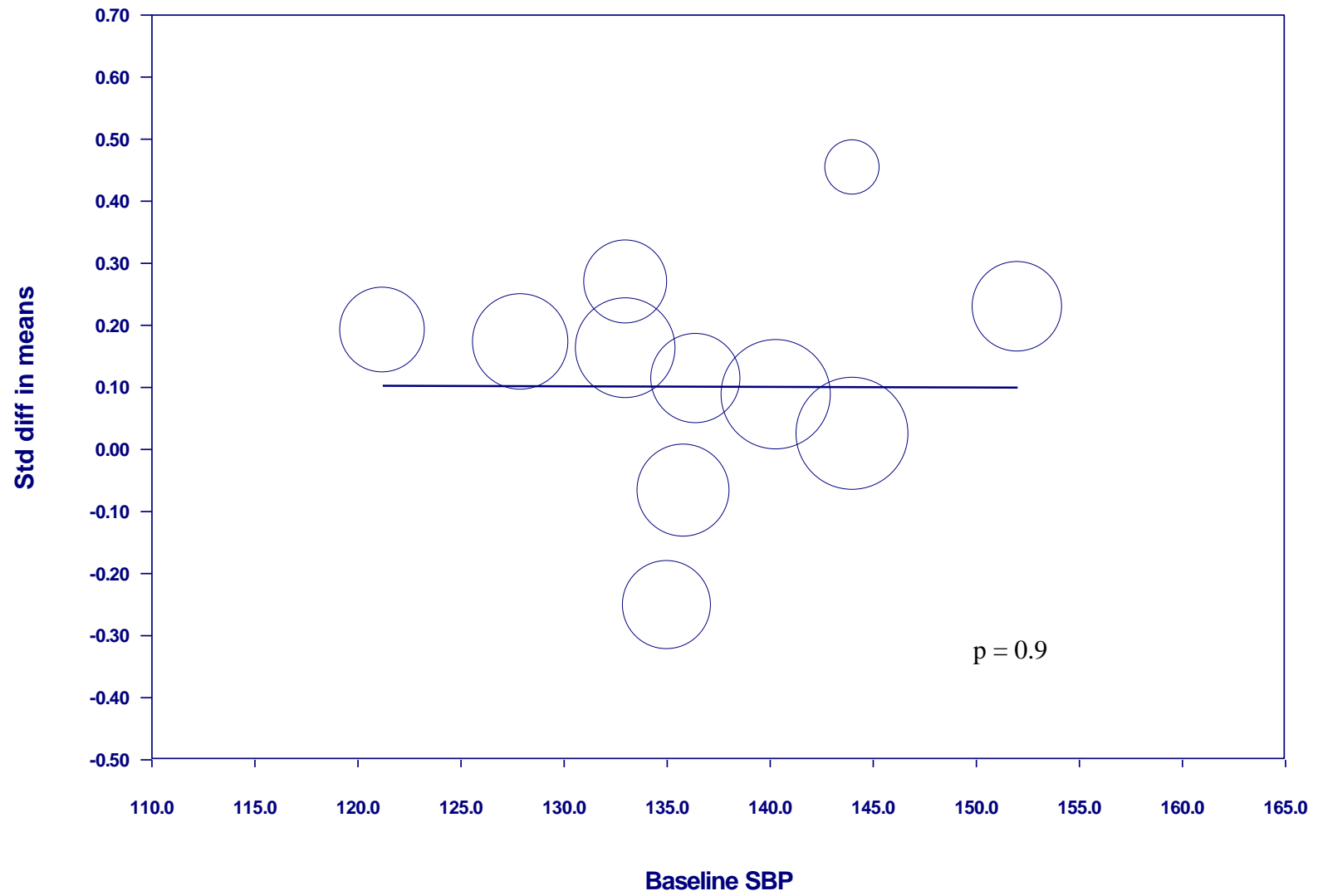

Supplement: Supplementary file 2 — Regression of standardized difference in means of included studies on baseline systolic blood pressure. (PDF 5 kb) [file 12889_2018_5326_MOESM2_ESM.pdf]

**Funnel Plot of Standard Error by Std diff in means**

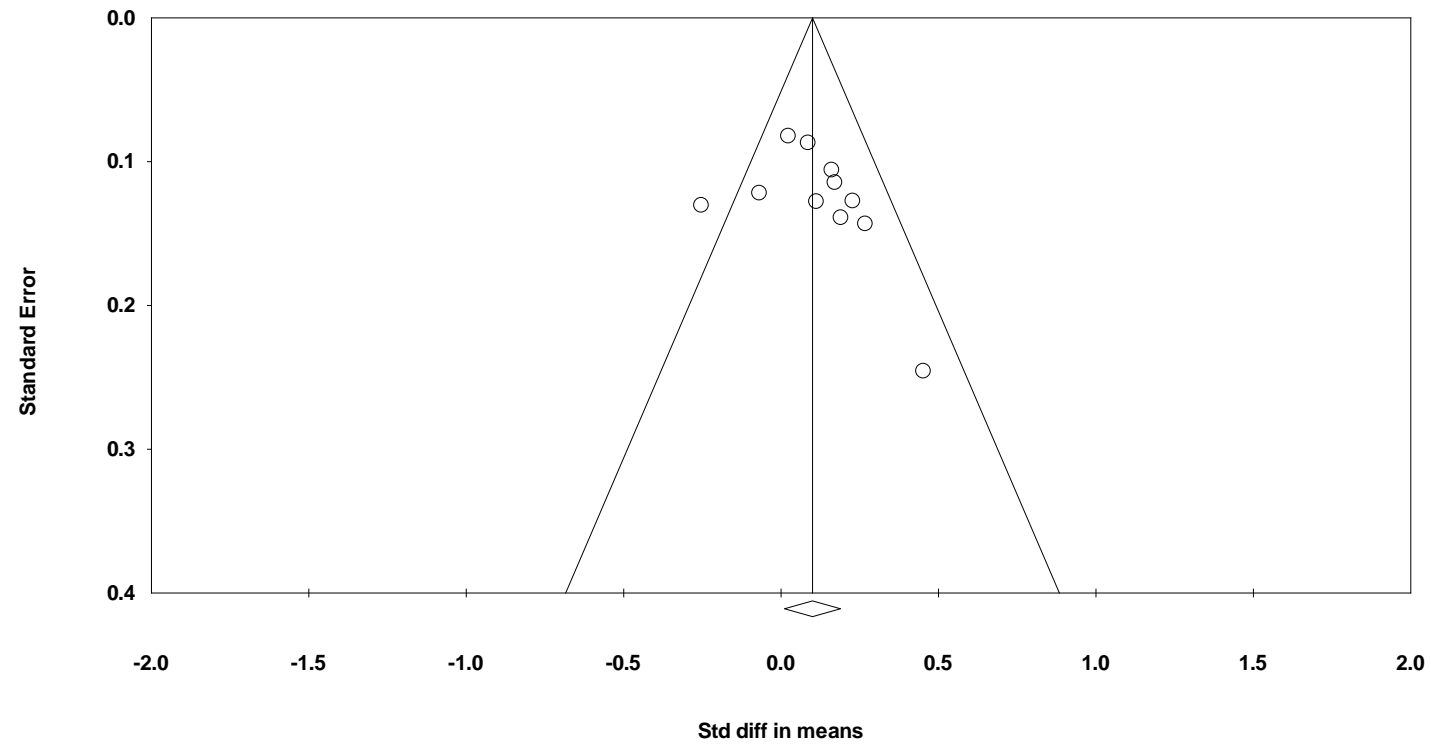

Supplement: Supplementary file 3 — Funnel plot of standard error by standardized difference in means for systolic blood pressure. (PDF 5 kb) [file 12889_2018_5326_MOESM3_ESM.pdf]

Regression of Std diff in means on Study duration in months

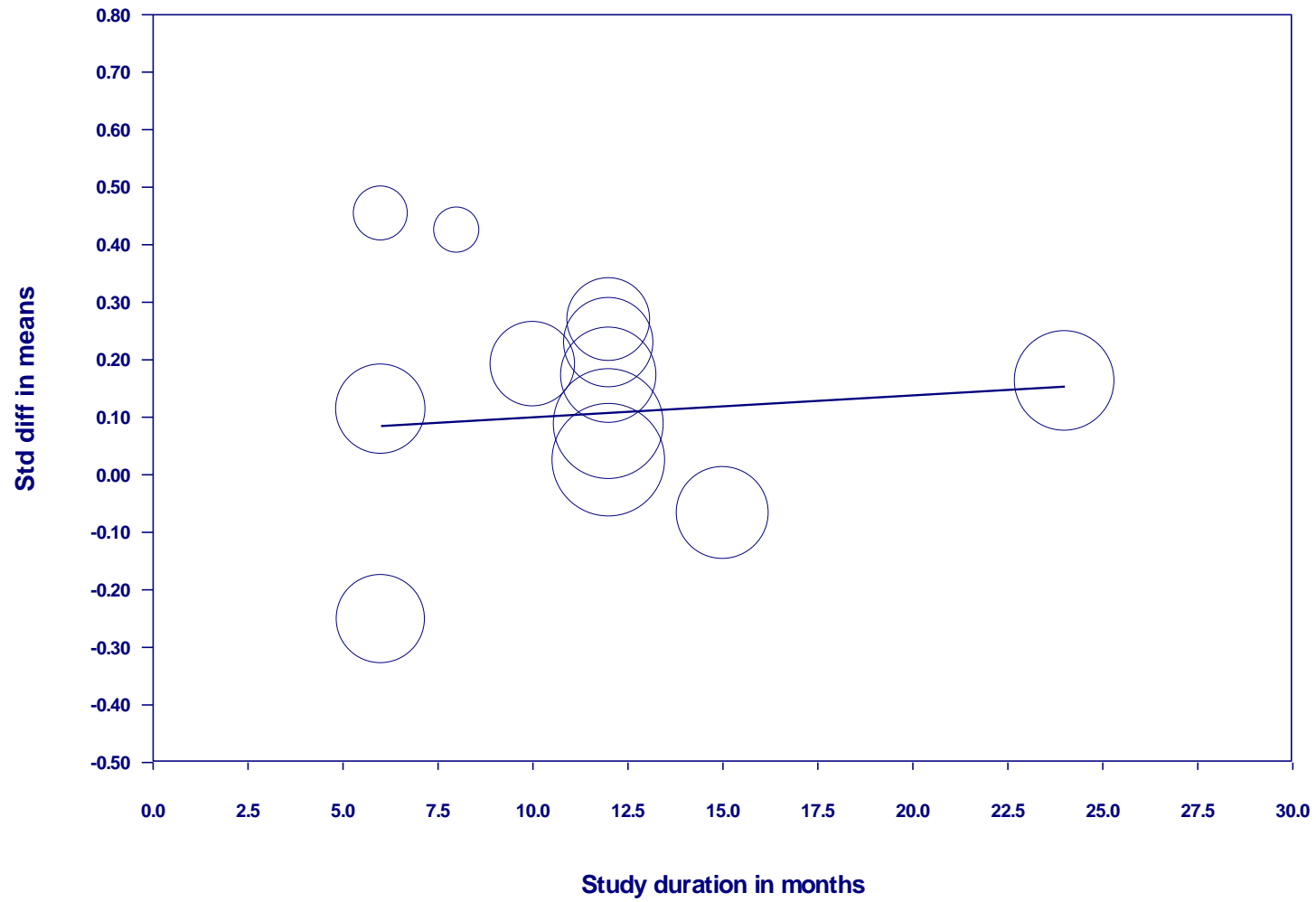

Supplement: Supplementary file 4 — Regression of standardized difference in means of included studies on Study duration. (PDF 84 kb) [file 12889_2018_5326_MOESM4_ESM.pdf]

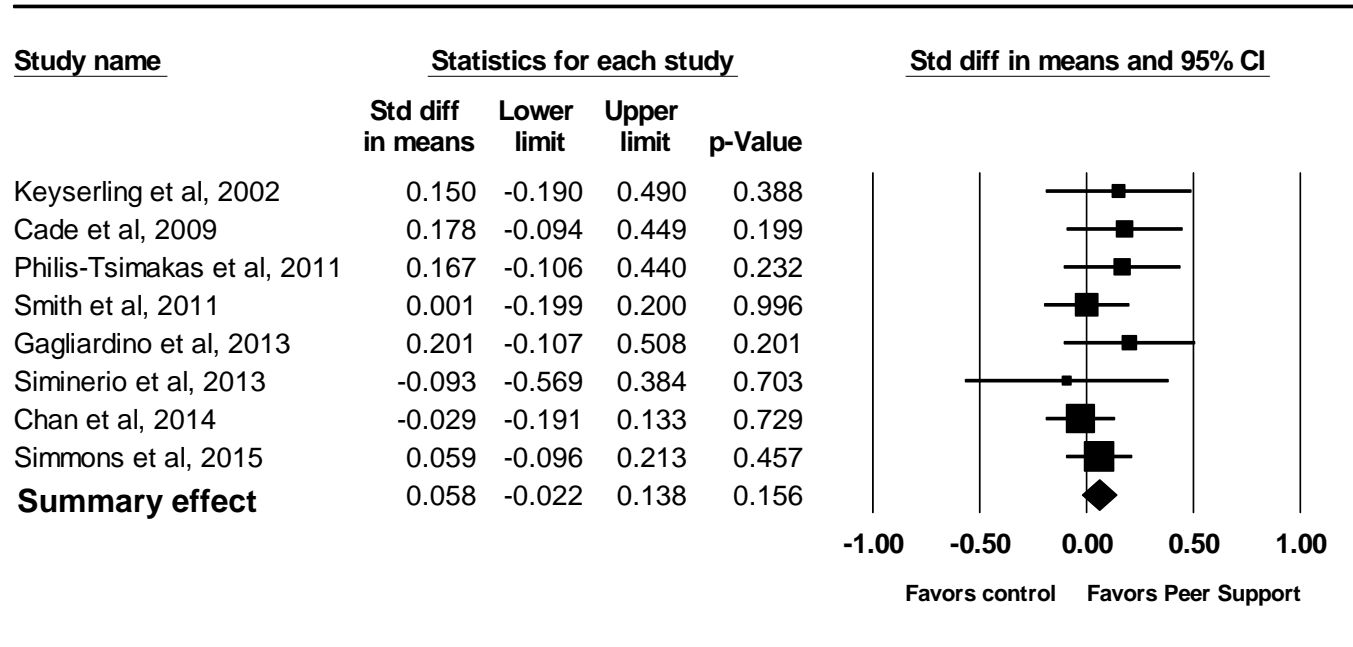

Supplement: Supplementary file 6 — Effect of peer support interventions on cholesterol in adults with diabetes. SMD = standardized mean difference; I2 0.00%, p for heterogeneity = 0.7. (PDF 8 kb) [file 12889_2018_5326_MOESM6_ESM.pdf]
