# Supplementary material for: Effect of peer support interventions on cardiovascular disease risk factors in adults with diabetes: a systematic review and meta-analysis
Source: BMC Public Health. 2018 Mar 23;18:398. doi: 10.1186/s12889-018-5326-8 (PMC5865386; doi:10.1186/s12889-018-5326-8)

Study name	Std diff in means	Lower limit	Upper limit	p-Value
Cade et al, 2009	0.075	-0.186	0.337	0.571
Heisler et al, 2010	0.053	-0.198	0.305	0.676
Philis-Tsimakas et al, 2011	0.340	-0.008	0.688	0.055
Gagliardino et al, 2013	-0.410	-0.696	-0.125	0.005
Siminerio et al, 2013	0.380	-0.100	0.861	0.121
Chan et al, 2014	-0.025	-0.187	0.137	0.764
Simmons et al, 2015	0.109	-0.062	0.280	0.213
Ayala et al, 2015	0.011	-0.231	0.253	0.930
Sazlina et al, 2015	0.084	-0.494	0.662	0.776
	0.039	-0.086	0.164	0.540

(Statistics for each study)

Std diff in means and 95% CI

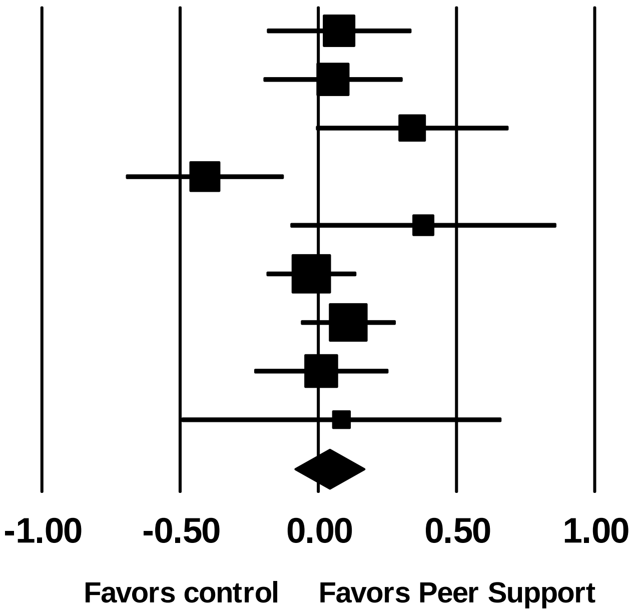

Supplement: Supplementary file 5 — Effect of peer support interventions on diastolic blood pressure in adults with diabetes. SMD = Standardized mean difference; Diastolic BP = systolic blood pressure. I2 49.07%, p for heterogeneity = 0.05. (PDF 8 kb) [file 12889_2018_5326_MOESM5_ESM.pdf]
