# Supplementary material for: Effect of peer support interventions on cardiovascular disease risk factors in adults with diabetes: a systematic review and meta-analysis
Source: BMC Public Health. 2018 Mar 23;18:398. doi: 10.1186/s12889-018-5326-8 (PMC5865386; doi:10.1186/s12889-018-5326-8)

Study name	Statistics for each study			
	Std diff in means	Lower limit	Upper limit	p-Value
Philis-Tsimakas et al, 2011	0.113	-0.236	0.463	0.525
Siminerio et al, 2013	-0.147	-0.624	0.330	0.545
Thom et al, 2013	0.027	-0.228	0.282	0.836
Chan et al, 2014	-0.036	-0.197	0.126	0.667
Safford et al, 2015	-0.114	-0.354	0.126	0.351
Sazlina et al, 2015	0.574	-0.016	1.164	0.056
**Summary effect**	-0.008	-0.124	0.109	0.895

Std diff in means and 95% CI

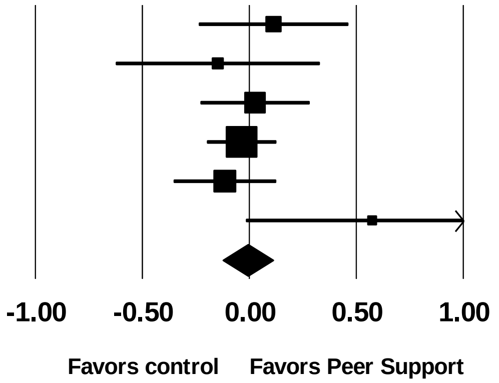

Supplement: Supplementary file 7 — Effect of peer support interventions on LDL cholesterol in adults with diabetes. SMD = standardized mean difference; I2 0.00%, p for heterogeneity = 0.819. (PDF 8 kb) [file 12889_2018_5326_MOESM7_ESM.pdf]
