# Supplementary material for: Effect of peer support interventions on cardiovascular disease risk factors in adults with diabetes: a systematic review and meta-analysis
Source: BMC Public Health. 2018 Mar 23;18:398. doi: 10.1186/s12889-018-5326-8 (PMC5865386; doi:10.1186/s12889-018-5326-8)

Study name	Std diff in means	Lower limit	Upper limit	p-Value
	**Statistics for each study**			
Cade et al, 2009	-0.018	-0.301	0.265	0.902
Philis-Tsimakas et al, 2011	-0.071	-0.344	0.201	0.608
Gagliardino et al, 2013	0.000	-0.286	0.286	1.000
Siminerio et al, 2013	-0.029	-0.505	0.447	0.905
Thom et al, 2013	0.000	-0.255	0.255	1.000
Chan et al, 2014	0.068	-0.094	0.230	0.410
Safford et al, 2015	-0.100	-0.340	0.140	0.414
McGowan et al, 2015	0.127	-0.136	0.390	0.343
Sazlina et al, 2015	0.276	-0.304	0.857	0.351
**Summary effect**	0.017	-0.071	0.104	0.706

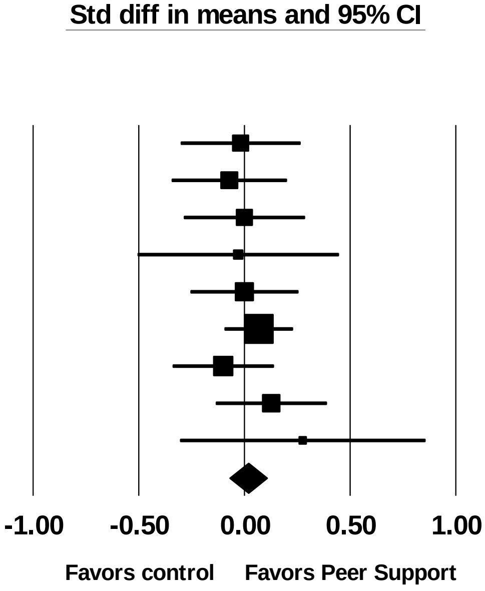

Supplement: Supplementary file 8 — Effect of peer support interventions on BMI in adults with diabetes. SMD = standardized mean difference; BMI = Body Mass Index; I2 0.00%, p for heterogeneity = 0.9. (PDF 86 kb) [file 12889_2018_5326_MOESM8_ESM.pdf]
