# Supplementary material for: Effect of peer support interventions on cardiovascular disease risk factors in adults with diabetes: a systematic review and meta-analysis
Source: BMC Public Health. 2018 Mar 23;18:398. doi: 10.1186/s12889-018-5326-8 (PMC5865386; doi:10.1186/s12889-018-5326-8)

Study name	Statistics for each study				Std diff in means and 95% CI
	Std diff in means	Lower limit	Upper limit	p-Value	
Keyserling et al, 2002	0.136	-0.240	0.512	0.479	
Lorig et al, 2008	0.015	-0.194	0.224	0.891	
Lorig et al, 2009	0.094	-0.172	0.360	0.489	
Smith et al, 2011	-0.029	-0.251	0.194	0.800	
Siminerio et al, 2013	-0.059	-0.535	0.418	0.809	
Chan et al, 2014	-0.026	-0.183	0.130	0.740	
Ayala, 2015	0.000	-0.225	0.225	1.000	
Sazlina et al, 2015	0.645	0.052	1.238	0.033	
**Summary effect**	0.019	-0.068	0.106	0.666	

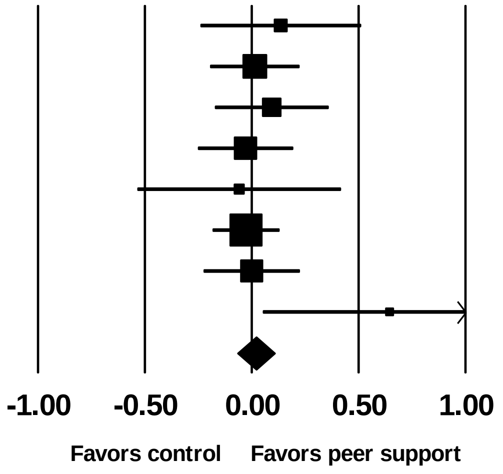

Supplement: Supplementary file 9 — Effect of peer support interventions on physical activity in adults with diabetes. SMD = standardized mean difference; I2 0.00%, p for heterogeneity = 0.47. (PDF 8 kb) [file 12889_2018_5326_MOESM9_ESM.pdf]
